# Molecular testing for cytologically suspicious and malignant (Bethesda V and VI) thyroid nodules to optimize the extent of surgical intervention: a retrospective chart review

**DOI:** 10.1186/s40463-021-00500-6

**Published:** 2021-04-28

**Authors:** Jessica Hier, Galit Avior, Marc Pusztaszeri, Joshua R. Krasner, Noura Alyouha, Veronique-Isabelle Forest, Michael P. Hier, Alex Mlynarek, Keith Richardson, Nader Sadeghi, Michael Tamilia, Richard J. Payne

**Affiliations:** 1 Faculty of Medicine, McGill University, 845 Rue Sherbrooke West, Montreal, QC Canada; 2 Department of Otolaryngology Head and Neck Surgery, Hillel Yaffe Medical Centre, Technion University, Hadera, Israel; 3 Department of Pathology, Sir Mortimer B. Davis-Jewish General Hospital, McGill University, Montreal, QC Canada; 4 Faculty of Pharmacology, McGill University, 845 Rue Sherbrooke West, Montreal, QC Canada; 5 Department of Otolaryngology Head and Neck Surgery, Sir Mortimer B. Davis-Jewish General Hospital, McGill University, Montreal, QC Canada; 6 Department of Otolaryngology Head and Neck Surgery, McGill University Health Centre, McGill University, Montreal, QC Canada; 7 Division of Endocrinology & Metabolism, Sir Mortimer B. David-Jewish General Hospital, McGill University, Montreal, QC Canada

## Abstract

**Background:**

Molecular testing has been used for cytologically indeterminate thyroid nodules (Bethesda III and IV), where the risk of malignancy is 10–40%. However, to date, the role of molecular testing in cytologically suspicious or positive for malignancy (Bethesda V and VI) thyroid nodules has been controversial. The aim of this study was to determine whether patients who had molecular testing in Bethesda V and VI thyroid nodules had the optimal extent of surgery performed more often than patients who did not have molecular testing performed.

**Methods:**

A retrospective chart review of 122 cases was performed: 101 patients from the McGill University teaching hospitals and 21 patients from the Hillel Yaffe Medical center, Technion University. Patients included in the study were those with Bethesda V or VI thyroid nodules who underwent molecular testing (ThyGenext® or ThyroseqV3®) (McGill *n* = 72, Hillel Yaffe *n* = 14). Patients with Bethesda V or VI thyroid nodules who did not undergo molecular testing were used as controls (McGill *n* = 29, Hillel Yaffe *n* = 7). Each case was reviewed in order to determine whether the patient had optimal surgery. This was defined as total thyroidectomy in the presence of either a positive lymph node, extrathyroidal extension, or an aggressive pathological variant of papillary thyroid carcinoma (tall cell, hobnail, columnar cell, diffuse sclerosing, and solid/trabecular) documented on the final pathology report. In all other cases, a lobectomy/hemi/subtotal thyroidectomy was considered as optimal surgery. Chi-squared testing was performed to compare groups.

**Results:**

When molecular testing was done, 91.86% (79/86) of surgeries in the molecular testing group were optimal, compared to 61.11% (22/36) in the control group. At McGill University teaching hospitals and at Hillel Yaffe, 91.67% (66/72) and 92.86% (13/14) of surgeries in the intervention group were considered as optimal, respectively. This compares to 58.62% (17/29) at McGill and 71.43% (5/7) at Hillel Yaffe when molecular testing was not performed (*p* = .001, *p* = .186).

**Conclusions:**

In this study, molecular testing in Bethesda V and VI thyroid tumors significantly improved the likelihood of optimal surgery. Therefore, molecular testing may have an important role in optimizing surgical procedures performed in the setting of Bethesda V and VI thyroid nodules. Prospective studies with larger sample sizes are required to further investigate this finding.

**Graphical abstract:**

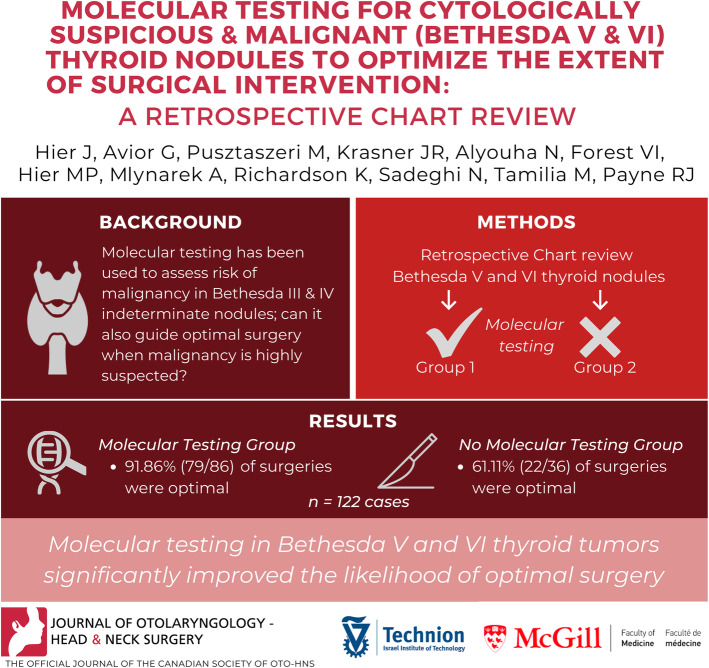

**Supplementary Information:**

The online version contains supplementary material available at 10.1186/s40463-021-00500-6.

## Introduction

Molecular testing has previously been used for cytologically indeterminate (Bethesda III and IV) thyroid nodules, where the risk of malignancy is 10–40% [1]. However, to date, there has been no established role for molecular testing in cytologically suspicious or positive for malignancy (Bethesda V and VI) thyroid nodules. According to the 2015 American Thyroid Association (ATA) Management Guidelines for Adult Patients with Thyroid Nodules and Differentiated Thyroid Carcinoma, after consideration of clinical and sonographic features, molecular testing may be considered in Bethesda V and VI thyroid nodules, if the result is expected to alter surgical decision making. According to some studies, preoperative clinical findings only manage to identify 18% of patients with indications for total thyroidectomy, and 53% of the remaining 82% of patients end up requiring a completion thyroidectomy [2].

Molecular signatures such as *BRAF V600E* and *TERT* have been shown to be associated with aggressive variants and advanced stage of papillary thyroid carcinoma [3]. *BRAF V600E* in particular has been shown to provide prognostic value well beyond the power of conventional clinicopathologic risk factors [4]. The 2015 ATA Management Guidelines for Adult Patients with Thyroid Nodules and Differentiated Thyroid Carcinoma incorporated several of these molecular mutations. Aggressive mutations include *BRAF V600E* and *TERT,* whereas other mutations such as *RAS, BRAF K601E* and *PAX8/PPARy* are considered to be of lower risk [3].

Total thyroidectomy is the recommended management for the majority of patients with well differentiated thyroid cancer (WDTC) where the risk of disease recurrence is intermediate or high. Unfortunately, the variables that designate a tumor as intermediate or high risk for recurrence are not often known prior to surgery. As a result, patients often undergo a hemi-thyroidectomy followed by a completion thyroidectomy. Failing to provide the optimal surgery at the onset wastes limited and precious health care resources and is detrimental to a patient’s well-being. The goal of this study was to determine whether patients with Bethesda V and VI thyroid nodules who underwent molecular testing, who did not have evidence of high-risk disease prior to surgery, had the optimal extent of surgery performed more often when compared to patients who did not have molecular testing performed. The significance of this study lies in its potential to provide clinicians with a better understanding of the association between molecular testing and clinical outcomes. Understanding this relationship is essential in improving the efficacy of thyroid cancer treatment and individualizing patient care.

## Methods & Materials

### Ethics

This study was approved by the Medical-Biomedical Research Ethics Committee (REC) of the Integrated Health and Social Services University Network for West-Central Montreal.

### Study design

A retrospective multicenter chart review of 122 medical charts was performed, 101 patients of which were treated at McGill University teaching hospitals and the remaining 21 patients were treated at the Hillel Yaffe Medical Center, which is affiliated with Technion University. Charts considered initially were patients with Bethesda V and VI thyroid nodules from 2018–2019. They were then included or excluded based on criteria described below. Patient characteristics including age, gender and tumor size were collected. Afterwards, all eligible patients were divided into two groups; those that underwent molecular testing and those that did not. The following data was collected from each patient and documented: fine needle aspirate (FNA) results (Bethesda V or VI, left or right nodule), if the patient had undergone molecular testing (ThyGenext® or ThyroseqV3®), if there was a mutation, the surgical procedure the patient underwent, and pertinent findings on the final pathology report. Each patient was categorized as having had or not had optimal surgical management according to the following criteria: total thyroidectomy was considered optimal if a positive lymph node (LN+) was found, if there was extrathyroidal extension (ETE), or if aggressive histologic variants of papillary thyroid carcinoma (tall cell, hobnail, columnar cell, diffuse sclerosing, solid/trabecular) were documented on the final pathology report. In all other cases, a lobectomy/hemi/subtotal thyroidectomy was considered optimal surgical management. At both sites, the extent of surgical intervention performed for each patient was guided by the 2015 ATA guidelines, however, the final decision in each case was determined on a case by case basis as per patient and surgeon preference.

### Patient selection

Patients included in the study were those with Bethesda V or VI thyroid nodules who underwent molecular testing (ThyGenext® or ThyroseqV3®) (McGill *n* = 72, Hillel Yaffe *n* = 14). Patients with Bethesda V or VI thyroid tumors who did not undergo molecular testing were used as controls (McGill *n* = 29, Hillel Yaffe *n* = 7). In order to target patients of intermediate risk according to the 2015 ATA guidelines, patients excluded were as follows: those with an aggressive variant of papillary thyroid carcinoma (tall cell, hobnail, columnar cell, diffuse sclerosing, solid and solid/trabecular) seen on initial FNA, patients that presented initially with metastatic disease (positive neck nodes), a nodule of > 4 cm, or a suspicious contralateral thyroid nodule.

### Sample collection

Informed consent was obtained from patients prior to undergoing an ultrasound-guided fine needle aspiration (USFNA). For those who agreed to undergo molecular testing, the specimen was transported to Interspace Diagnostics in Pittsburgh for ThyGenext® or to a commercial laboratory at the University of Pittsburgh Medical Centre in Pittsburgh for ThyroseqV3®. The final surgical pathology specimens were reported by thyroid pathologists who have substantial experience commenting on aggressive features such as tumors with ETE, LN+, and the following variants of papillary thyroid carcinoma: tall cell, hobnail, columnar cell, diffuse sclerosing, and solid/trabecular.

### Statistical analysis

Chi-square testing was performed to compare the proportion of patients who had optimal management in those that did undergo molecular testing and those that did not. Significance was confirmed at a *p*-value of < 0.05.

## Results

### Patient characteristics

One hundred one charts from McGill University teaching hospitals and 21 charts from the Hillel Yaffe Medical Center, Technion were included in this study. Patient characteristics for both patients that underwent molecular testing and patients that did not, were gathered and summarized in Table 1. Characteristics considered were: age, gender, U/S measured nodule size and Bethesda score.

#### Baseline patient characteristics

**Table 1:** Baseline patient characteristics at McGill, at Hillel Yaffe and when data was pooled. No statistically significant differences were found between the groups in terms of age, gender distribution, U/S determined nodule size or Bethesda score (*p* > 0.05 for each category at both centres, 95% CI)

	Control	Molecular Testing
**Age (yrs)**	54.1 (48.4–59.8)	49.2 (46.0–53.5)
McGill	52.3 (45.8–58.7)	48.7 (45.3–52.1)
Hillel Yaffe	61.7 (47.2–76.3)	51.9 (41.2–62.2)
**Female gender (%)**	80.3	86.8
McGill	74.9	73.6
Hillel Yaffe	85.7	100
**Nodule size (cm)**	1.9 (1.5–2.2)	1.8 (1.6–2.0)
McGill	1.9 (1.6–2.2)	1.8 (1.6–2.0)
Hillel Yaffe	1.4 (0.9–2.5)	1.6 (1.3–2.2)
**Bethesda Score of VI (%)**	52.8	48.8
McGill	44.8	45.8
Hillel Yaffe	64.3	85.7

### Molecular testing and optimal surgery

**Fig. 1:**
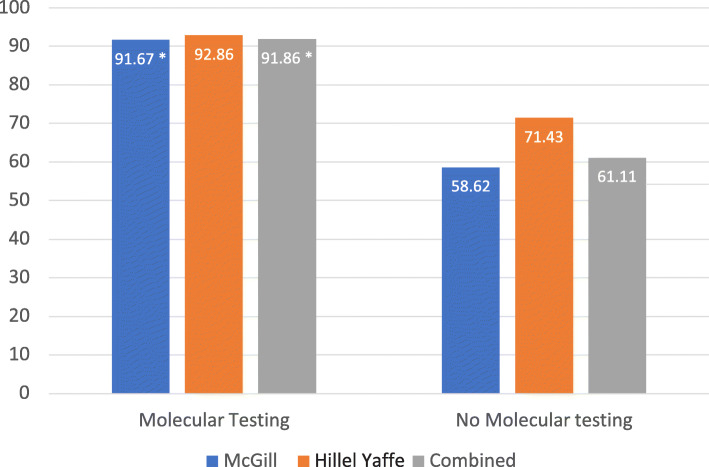
Percentage of cases that had optimal surgery in the molecular testing and non-molecular testing groups. * = significant (*p* < 0.05)

Each chart was reviewed and was categorized as having been handled optimally or not based on the clinical criteria mentioned previously. The data is summarized in Table 2. Figure 1 depicts that a statistically significant difference was found between the intervention and control group at McGill and when the data was pooled.

#### Molecular testing and optimal surgery

**Table 2:** Correlation between molecular testing and optimal surgical intervention at McGill, Hillel Yaffe and when data was pooled. The relationship between molecular testing and optimal surgery proved to be statistically significant at McGill and with pooled data. *P* value < 0.05

	Control (*n* = 36)	Molecular Testing (*n* = 86)
Optimal Surgery (%)	61.1	91.9
McGill	58.6 (*n* = 29)	91.7 (*n* = 72)
Hillel Yaffe	71.4 (*n* = 7)	92.9 (*n* = 14)

#### Molecular testing and optimal surgery (%)

The mutations found in the intervention group are seen below in Fig. 2. The most predominant mutation found was *BRAF V600E*, a well-documented predictor of aggressive disease [5]. The second most common occurrence was that no mutation was present. Other mutations encountered were: *TERT, PET/PTC6, HRAS, PAX8/PPARy, KRAS, NRAS, BRAF K601E, PTEN*.

### Mutation distribution

**Fig. 2:**
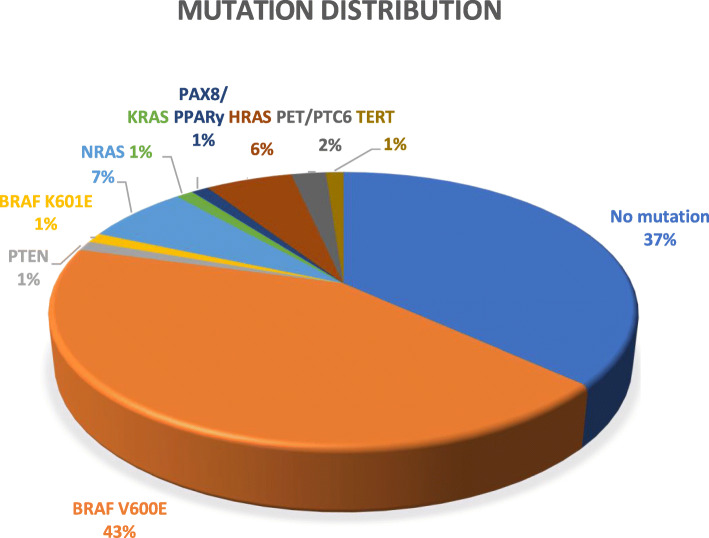
Mutation distribution in the intervention group, using ThyGenext® or ThyroseqV3®. The most prevalent mutation type was BRAF V600E, a high-risk mutation. *The one case with a TERT mutation had a co-mutation of BRAF V600E

## Discussion

To understand the clinical importance of this study, one must consider the repercussions of both over and undertreatment of patients with WDTC. With undertreatment, patients are subjected to a second surgery, which brings about more physical and psychological repercussions. Additionally, more healthcare system resources must be put towards treatment, surgery wait times increase, and patients must take more time off work.

Overtreatment in this case would be a total thyroidectomy when a hemi-thyroidectomy may be more appropriate. This leads to a lifelong dependence on thyroid hormone replacement therapy and the increased risk of important complications associated with a total thyroidectomy, such as vocal cord paralysis and hypoparathyroidism. For these reasons, there are significant benefits to the patient and health care system when a patient receives the optimal initial surgical management for WDTC. The population considered in this report are patients with cytologically suspicious or malignant thyroid nodules exhibiting an intermediate risk profile according to the 2015 ATA guidelines. The study was designed this way as these are the patients in which molecular testing has the greatest potential to alter management in either direction.

Clinical decision making in WDTC is challenging in patients with Bethesda V and VI thyroid nodules that measure between 1 and 4 cm and that lack accepted aggressive features. Therefore, determining the level of risk of recurrence of the disease and by extension, the appropriate management is unclear [4]. For this reason, Xing et al. looked at the association of the *BRAF V600E* mutation and the recurrence of papillary thyroid cancer. They showed that this mutation has an independent prognostic value for WDTC recurrence and therefore has potential value in preoperative decision-making. Another group, Krasner et al. evaluated the association between WDTC molecular mutations and aggressive characteristics to assess the usefulness of molecular mutations in determining extent of thyroid surgery. It was found that BRAF V600E, RET/PTC1 and TERT mutations were associated with aggressive characteristics, whereas BRAF K601E, HRAS, NRAS, KRAS, PAX8-PPARy mutations and tumors with no mutations are significantly less likely to be associated with high risk cancers. This study demonstrated that molecular testing is likely a valuable preoperative tool to help guide surgical management of thyroid cancer [6].

Prior to the 2015 ATA guidelines, the recommended therapy for thyroid cancer was a total thyroidectomy. In 2015, physicians were given a choice; according to the 2015 ATA guidelines, “for patients with thyroid cancer >4 cm, or with gross extrathyroidal extension or clinically apparent metastatic disease to nodes or distant sites, the initial surgical procedure should include a near-total or a total thyroidectomy” [3]. This change in the guidelines resulted in more hemithyroidectomies being done, and consequently, more completion surgeries as well. This study was conducted because there is a newfound need for a prognostic tool that permits greater physician accuracy and thereby, more efficacious patient care. Our team sought to gather data from two different institutions, to determine whether molecular testing in Bethesda V and VI thyroid nodules leads to optimal surgery when compared to those that did not undergo molecular testing.

In keeping with our initial hypothesis, patients who had undergone molecular testing (ThyGenext® or ThyroseqV3®) had experienced higher rates of optimal surgical management, both at McGill and at Hillel Yaffe. At McGill, statistical significance was present, whereas at Hillel Yaffe, although a greater proportion of patients that had undergone molecular testing had optimal surgery, there was no statistical significance shown. The main limitation in this setting was sample size, having had only fourteen test subjects and seven controls.

In an attempt to quantify the usefulness of molecular testing in Bethesda V and VI nodules of low to intermediate risk of recurrence, we also looked at how frequently molecular testing succeeded at changing the initially planned management. When the data was pooled for the two participating centers, those who received molecular testing as part of their preoperative work-up experienced optimal surgical management in 91.9% of the cases, compared to 61.1% of the cases in the control group. From this, we see that over 30% more patients received the optimal treatment when molecular testing was included in the management decision-making process.

Within the molecular testing group, the extent of surgery was altered in 21 of 86 cases (24.4%) when using the aforementioned definition of optimal surgery. This was made up of two groups, 15 of which (17.4% of the molecular testing cohort) had an appropriate increase in extent of surgery (preventing undertreatment) whereas 7 patients (8.1% of the molecular testing cohort) had an appropriate decrease (preventing overtreatment). Using this tool to individualize treatment resulted in significantly fewer patients receiving non-optimal treatment.

Our findings were similar to those found in the literature. Yip et al. performed a cohort study in which 671 patients with differentiated thyroid cancer were divided into a molecular testing group and a control group, in which no molecular testing was performed. The goal of their study was to determine if routine cytological molecular testing promotes total thyroidectomy for clinically significant thyroid cancer and limits surgery to lobectomy when appropriate. Their results showed that without molecular testing, patients who received initial lobectomy were 2.5 times more likely to require a completion surgery [7]. Another group, McCoy et al. performed a retrospective cohort study with 670 patients, divided into a molecular testing and a non-molecular testing group. In both cohorts, intra-operative pathologic examination was performed during lobectomy and findings suggestive of aggressive disease prompted a total thyroidectomy. Preoperative molecular testing allowed for an increased rate of initial total thyroidectomy, eliminating the need for a later completion [8]. Our study, however, was the first to look specifically at Bethesda V and VI nodules.

In our study, the most common result of molecular testing was a *BRAF V600E* mutation (43% of cases, Fig. 2), which is comparable to previous literature. In a meta-analysis of 3437 patients performed by Li et al., 47.48% of thyroid nodules tested positive for *BRAF V600E* [9]. The second most frequent outcome in our study was the absence of a mutation (37% of cases, Fig. 2). These two outcomes accounted for 80% of the cases in the intervention group, both of which provide an important piece of information that can aid in optimizing management. Other mutations that present in a minor subset of cases were *TERT, PET/PTC6, HRAS, PAX8/PPARy, KRAS, NRAS, BRAF K601E, PTEN. TERT* promoter mutation is a high-risk molecular signature for disease recurrence, whereas the other mutations listed are of low to moderate risk [3]. In one case, both *TERT* and *BRAF V600E* mutations were found, which together predict PTC-specific mortality beyond the prognostic abilities of classic clinicopathologic factors [10]. In this study, ThyGenext® was used much more frequently than Thyroseq V3® (82/86 cases), therefore no conclusions can be drawn as to whether one test provided an increased rate of optimal surgical management over the other. It is worth noting that patients with Bethesda V or VI thyroid nodules were offered molecular testing at the treating specialist’s discretion. Therefore, different specialists made their own decision on when molecular testing was appropriate. Additionally, the choice between tests (ThyGenext® and Thyroseq V3®) was made in accordance with both specialist and patient preferences. The subjective nature of this choice demonstrates the need for clear molecular testing guidelines.

Molecular testing expenses were not covered by insurance and as a result a certain proportion of patients had the test covered by their provincial health care plan, while others had to pay out of pocket. Therefore, selection bias lies in the fact that in general, patients receiving this test had the financial means to pay for it. One could thereby argue that the patients who underwent molecular testing have a better baseline health status than the control group patients. However, this is of little consequence to the current study, as the decision of the appropriateness of surgery was individualized to each patient’s nodule characteristics.

A possible confounding variable to consider is the relationship between those who opted for genetic testing and their likelihood to opt for more aggressive surgery. Within our data set, there was only one patient who made a decision that opposed the specialist’s recommendations, which were based on clinicopathologic findings as well as molecular testing. This patient was categorized as a case of non-optimal surgery within the molecular testing group.

The limitations of this study are inherent to a study conducted in a retrospective manner. In an effort to address these limitations, inclusion and exclusion criteria were carefully designed in order to minimize the retrospective bias. Also, the reporting of features in imaging modalities, ultrasound in this case, can be subject to interobserver variability as the data from multiple physicians was used.

## Conclusion

In conclusion, our study demonstrates a positive correlation between molecular testing for Bethesda V and VI thyroid nodules and the likelihood of optimal extent of surgery, meaning that patients who underwent molecular testing were more likely to undergo optimal surgery. This relationship was statistically significant. This suggests that molecular testing may play an important role in the preoperative decision-making process for patients with Bethesda V and VI thyroid nodules. Long-term prospective studies with larger sample sizes, cost-effectiveness studies and studies comparing the different molecular testing modalities are required to determine whether there is a role for molecular testing in nodules with a high cytologic probability of thyroid cancer, rather than solely in cytologically indeterminant thyroid nodules.

## Supplementary Information

**Additional file 1.**

## Data Availability

All data generated or analysed during this study are included in this published article [and its supplementary information files].
